# Discovering Daily Activity Patterns from Sensor Data Sequences and Activity Sequences

**DOI:** 10.3390/s21206920

**Published:** 2021-10-19

**Authors:** Mirjam Sepesy Maučec, Gregor Donaj

**Affiliations:** Faculty of Electrical Engineering and Computer Science, University of Maribor, Koroška Cesta 46, SI-2000 Maribor, Slovenia; gregor.donaj@um.si

**Keywords:** activities of daily living, sensors, Hamming distance, clustering, entropy

## Abstract

The necessity of caring for elderly people is increasing. Great efforts are being made to enable the elderly population to remain independent for as long as possible. Technologies are being developed to monitor the daily activities of a person to detect their state. Approaches that recognize activities from simple environment sensors have been shown to perform well. It is also important to know the habits of a resident to distinguish between common and uncommon behavior. In this paper, we propose a novel approach to discover a person’s common daily routines. The approach consists of sequence comparison and a clustering method to obtain partitions of daily routines. Such partitions are the basis to detect unusual sequences of activities in a person’s day. Two types of partitions are examined. The first partition type is based on daily activity vectors, and the second type is based on sensor data. We show that daily activity vectors are needed to obtain reasonable results. We also show that partitions obtained with generalized Hamming distance for sequence comparison are better than partitions obtained with the Levenshtein distance. Experiments are performed with two publicly available datasets.

## 1. Introduction

The number and proportion of elderly people in the population are increasing. In 2019, the number of people aged 60 years and older was 1 billion. This number will increase to 1.4 billion by 2030 and 2.1 billion by 2050 (https://www.who.int/health-topics/ageing#tab=tab_1, accessed on 1 August 2021). The world’s aging population is placing increasing pressure on health and social systems, and healthcare providers are struggling to care for elderly people efficiently. In addition, the cost of caring for the elderly in nursing homes is much higher than the cost of in-home care. All these facts forced the fast development of new technologies that can help seniors to stay at home and remain independent for longer [1,2]. Smart home environments are environments that attempt to make the life of their residents more comfortable by using technology that monitors the residents’ activities. Monitoring can be performed using video cameras—these approaches are called vision-based approaches [3]. They are problematic with regard to the security and privacy concerns of the residents. The alternative is sensor-based approaches, in which home environments are equipped with several sensors and smart devices. Sensors gather information of different types. The approaches differ based on sensor deployment, which can be wearable or environmental [4,5]. The major problem with wearable sensors is that wearing a tag is sometimes not feasible [6].

For example, in the case of elderly persons or patients, they may forget to wear the tags or they may resist wearing the tags at all. On the other hand, environmental sensors are attached to objects in a house or apartment, and the resident does not need to care about them, except for occasional battery changes. Environmental sensors have many advantages, such as being low cost, less intrusive, and more privacy-preserving [5].

Activity recognition infers a person’s activities from monitoring the environment. Approaches that recognize activities from simple environment sensors have been shown to perform well with an accuracy exceeding 90% [7,8,9]. However, the problem lies in how to interpret the gathered information and what to infer from it. The goal is to detect changes in the activities of daily living (ADL) as they might indicate deteriorating health or mental condition [10]. The ability to detect an emergency situation and set off an alarm is crucial in such environments.

Two commercial solutions are available today: A wearable alarm button to call for help and wearable systems based on accelerometers for automatic fall detection. Such systems require the user to be involved actively by wearing the device, pushing the button, charging the batteries, etc. Automated detection of the unusual behavior of the resident could assist in earlier diagnosis of physical or mental decline and timely treatment. However, the high level of complexity in activity patterns and a large amount of noise stemming from real-life behaviors pose great challenges in achieving this task.

What is unusual behavior of a resident? It is behavior that deviates from their routine [11]. For example, if a resident leaves home frequently but suddenly is at home almost all the time, it could indicate social isolation. On the contrary, if a resident hardly ever leaves home and suddenly the frequency of leaving and returning home increases, it could indicate dementia. Another example is a significant change in personal hygiene practices. For example, if we notice that a resident is bathing infrequently, but usually he was bathing frequently and for a long time, this could indicate a fear of falling in the shower or bath.

Some behavioral patterns could be typical for one person and unusual for another, or could be typical for weekdays and unusual for weekends. For this reason, we define our research problem to discover several different usual behavior patterns of a resident. Our starting point is the claim that a resident, whose activities are recorded in the dataset, is healthy and behaves normally. We define usual behavior patterns as partitions in a clustering algorithm used with recorded data. Later, changes in those patterns, such as frequent new patterns that do not fit in any partition, could be declared as unusual and may be indicators of declining health.

The remainder of the paper is organized as follows. In Section 2, we present an overview of related work. In Section 3, we detail the descriptions of two basic metrics for sequence comparison. Section 4 presents the proposed framework, which consists of a newly proposed sequence comparison and clustering. First, mathematical definitions are given for different comparisons of sensor sequences and activity sequences. Afterward, the clustering method is explained, based on proposed comparisons. Section 5 presents the results of our experiments. We conclude the paper with a final discussion in Section 6.

## 2. Related Work

Due to remarkable improvements in sensor technology, interest in activity recognition has increased significantly in the last decade [5,12]. Recently, ADL recognition systems were proposed that utilize the sensor data from smartphones [13,14]. There are three main groups of approaches for sensor-based activity recognition: Data-driven, knowledge-driven, and hybrid approaches. Data-driven approaches use various machine learning techniques to learn activities from collected sensor data. The most frequently used are: Naive Bayes classifier [15], Hidden Markov Models [8,16,17], Support Vector Machines [3], dictionaries of patterns [18], and neural networks [6,19,20]. These approaches require a great amount of annotated data to train the models accurately.

For that reason, the scientific community has developed and provided a considerable amount of data sets [21]. The idea of knowledge-driven approaches is to use prior knowledge to create rough activity models. Ontology-based activity recognition was shown to perform comparably well to the data-driven approaches [22]. Hybrid approaches take advantage of the positive features of data-driven and knowledge-driven approaches [23].

Supervised training poses a problem when applying the models on a large scale. Due to differences in monitored environments, a model trained in one environment cannot be used for another environment. Transfer learning was studied to avoid the need to gather a labeled dataset for each new environment [17]. The use of a prior distribution over the model parameters has proved to be efficient with probabilistic models. The prior distribution provides an initial estimate of the model parameters for the target environment and is learned from the source environment. The influence of the prior distribution decreases as more training data are observed for the target environment.

Another problem in ADL recognition is unseen activities. Machine learning algorithms classify activities whose instances have already been seen during training. Very recently, zero-shot learning methods were proposed, which can extend the learning model to detect unseen activities without prior knowledge regarding sensor readings about those previously unseen activities [24].

A literature review has shown that many problems regarding ADL recognition were addressed, and the proposed solutions demonstrated good results [7,8,9]. However, the question remains what to infer from the recognized sequence of activities. Clustering the sequences could identify typical types of patterns in activity sequences. Noticing unusual patterns of activity sequences could indicate changes in a person’s behavior.

Clustering and sequence comparison is studied widely in Bioinformatics, where similarity between protein sequences is sought in order to cluster them into groups of sequences with similar functionality or structure [25]. Biological sequences are greatly different from activity sequences with respect to the timing and duration of the sequence elements. Sequence analysis is also a key method in Social Sciences, where it is used to study the spans of life trajectories and careers [26,27].

Measuring the similarity between sequences depends highly on the choice of similarity measure. Different measures were studied in [28,29]. The first group of analyzed measures is based on distances between probability distributions. The second is based on counts of common attributes, and the third group of measures looks for optimal matching between sequences [30]. In the framework of ADL, we expect the measure to reflect differences in the timing, duration, and sequencing. From the theoretical knowledge, no measure dominates all others in all three dimensions of interest.

Discovering the ADL patterns performed in a day has been a relatively unexplored research area. Activities were discovered by clustering [7]. They employed activity clustering to group the patterns into activity definitions, where the partition centroids represented the activities that were tracked and recognized afterwards. The k-nearest neighbors algorithm is the most widely used clustering algorithm for ADL recognition [4]. In [31], a two-stage ADL recognition was defined, where, in the first stage, activity records were clustered into two partitions by regarding temporal features, and, in the second stage, the classifiers were used to recognize the daily activities in each partition according to the spatial features. Recently, a self-organizing neural network model was presented that considers the following ADL features: The ADL start time, duration, and spatial information [32].

Until recently, research works were focused on ADL recognition with the aim to increase the accuracy of recognition results [33]. However, these works did not analyze recognized activities to determine behavior patterns. Contextual behavior patterns were studied in [34]. Context features were the day of the week, weather, season, noise levels, visitor presence, etc. In [11], normal behavior patterns were defined as lists of activities that a resident performs in their house, with the time of the day and duration. Lists were made from recorded data. Deviations from those definitions were discovered by a decision-support system and may indicate unusual behavior.

In [35,36], an activity-dependent anomaly detection approach was defined, and “sleeping” was selected as the activity of interest. As data similarity measures, Euclidean, Chebyshev, and Canberra distances were studied. A literature review demonstrated that behavior patterns always corresponded to time intervals of one activity.

A summary of selected references from the reviewed literature is presented in Table 1.

### Aim and Research Contribution

This paper aims to analyze a person’s daily activities that are usual and to identify their patterns. Our proposed framework differs from the research in the literature in that we are looking for patterns based on activity vectors of whole days. In contrast, in the research work in the literature, methods were examined for pattern extraction from shorter interval sequences [7]. Consequently, clustering of the extracted sensor data patterns is studied in the literature, where the obtained clusters define single activities. In our research, clusters present groups of activity patterns for whole days. Most related research is aimed toward correct ADL recognition, whereas our research aims to use the recognition results as a starting point for discovering the usual behavior of residents.

The research contribution of the present study is the definition of simple similarity metrics, adapted to vectors of sensor data and vectors of daily activities, and the application of clustering to both types of vectors, with the idea to identify days with similar patterns of resident behavior. Such partitions could be used to detect days with unusual patterns of activities.

Our similarity metrics differ from those in the literature in certain aspects. First, they are applied to vectors representing whole days with one entry for one second. The exception are vectors used in the case of the Levenshtein distance. Those vectors are shorter, with one entry for one activity in the sequence. Similarity metrics in literature are derived from numerical distances, whereas our aim was to define a metric applicable to original data. The essential difference in vector comparison is also its sensitivity to the adjacency of activities.

Clustering in literature is applied to specific activity records or is used in the scope of ADL recognition [4]. We apply clustering to ADL sequences representing whole days.

Previous research works do not analyze all activities of the resident performed during the day. They mainly focus on the behavior changes related to one activity only. For example, [35] focuses on the behavior changes related to sleeping, whereas the authors in [37] developed the solution to quickly detect “a fall” of the monitored person.

## 3. Preliminary

We chose two distance metrics in our research. The Hamming distance was chosen because it is the basic metric for comparing sequential data of equal length. We can use it to compare full-length sensor and activity data.

To compare daily activity vectors, we may also consider that the duration of activities may vary. If we later discharge this duration by merging repetition of the same activity, the Hamming distance cannot be used, because the vectors are now shorter and not of the same length anymore. The Levenshtein distance can be used instead, to determine if two vectors could be considered variations of the same pattern. If a resident would shift his daily routine, such as waking up later, the Levenshtein distance would not be affected, since the sequence of activities would not change.

We wanted to compare these two distances to find which metric was more appropriate for detecting unusual behavior.

### 3.1. Hamming Distance

In general, the Hamming distance between two vectors $\vec{x}$ and $\vec{y}$ is the number of positions in which the two vectors are different:

(1) $$H\left(\vec{x},\vec{y}\right)=\sum_{i=1}^{n}\mathrm{diff}\left(x_{i},y_{i}\right),$$

where *n* is the dimension of the vectors, $x_{i}$ and $y_{i}$ are the *i*-th components of vectors $\vec{x}$ and $\vec{y}$, respectively. The difference function diff gives a result of 1 if $x_{i}$ and $y_{i}$ differ, and 0 if they are the same. This distance can only be applied to sequences of equal length.

In order to use the Hamming distance in our research, we needed to make some generalizations to the diff function, which we present in the next section.

### 3.2. Levenshtein Distance

The Levenshtein distance is given by the smallest number of edit operations needed to turn one sequence into another. The Levenshtein distance between two vectors $\vec{x}$ and $\vec{y}$ is defined as:

(2) $$L\left(\vec{x},\vec{y}\right)=\left\{\begin{array}{cc} |\vec{x}|\cdot cost_{D}, & \mathrm{if}\;|\vec{y}|=0,\\ |\vec{y}|\cdot cost_{I}, & \mathrm{if}\;|\vec{x}|=0,\\ L(tail\left(\vec{x}\right),tail\left(\vec{y}\right)), & \mathrm{if}\;x_{1}=y_{1},\\ min\left\{\begin{array}{c} L\left(tail\left(\vec{x}\right),\vec{y}\right)+cost_{D},\\ L\left(\vec{x},tail\left(\vec{y}\right)\right)+cost_{I},\\ L\left(tail\left(\vec{x}\right),tail\left(\vec{y}\right)\right)+cost_{S} \end{array}\right. & \mathrm{otherwise}. \end{array}\right.$$

Here, $|\vec{x}|$ denotes the length of vector $\vec{x}$, and $tail(\vec{x})$ is vector $\vec{x}$ without the first element. Edit operations (insertion, deletion, or substitution) are penalized by costs (i.e., $cost_{I}$, $cost_{D}$, or $cost_{S}$), which are all equal to one in the original version of the distance. As one substitution equals one deletion and one insertion, its cost could be the sum of the cost of deletion and the cost of insertion. Using more than one substitution cost allows even more flexibility in the comparison.

## 4. Proposed Framework

Our research framework addresses the problem of discovering the daily activity patterns of the resident. Identifying patterns is difficult, as the duration and the order of activities may vary from one occurrence to another. In some pattern occurrences, certain activities may be missing.

**Figure 1:**
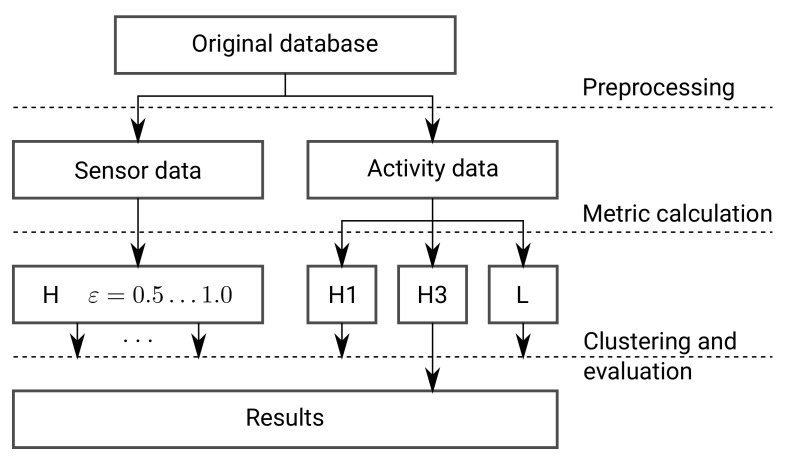
Flowchart of the proposed framework.

The proposed framework is based on the premise that time annotated sensor readings and daily activity sequences of a resident are available. The entire framework is presented in Figure 1 and is composed of five tasks: Data preprocessing, comparing sequences by metric calculation on sensor and activity data separately, clustering based on pairwise comparison methods with evaluation of the clustering results, visual representation of clusters, and visual representation of daily activity vectors within clusters. The first task, data preprocessing, generates a set of vectors of active sensors and a set of vectors of daily activities. This task is described in the next section. The second task, comparing sequences, provides definitions of distance metrics, adapted to sensor data and activity sequences, respectively. The third task, clustering, provides clusters of similar vectors based on distance metrics. These two tasks are described in the continuation of this section. The fourth and the fifth tasks visualize the results of clustering. They are given in the next section. The visual representation of daily activities within clusters shows similar daily activity patterns of the resident.

### 4.1. Comparing Sequences of Active Sensors

The basic source of information about a resident’s activities is sensor data. The sensor data stream is organized into a vector of active sensors sets over time:

(3) $$\vec{s}=\left[s_{1},\ldots ,s_{n}\right],\;s_{i}\subset S,$$

where S is a finite alphabet of sensors, and $s_{i}$ is a set of sensors active in time slot *i*. The dimension of the vector *n* depends on the time scale. For example, if time slots correspond to seconds, and the vector corresponds to a full day, the dimension *n* is 86,400.

Considering vectors of active sensors $\vec{s}$ and $\vec{q}$, at each time slot *i*, we have sets of active sensors $s_{i}$ and $q_{i}$. In this case, we define the difference function as:

(4) $${\mathrm{diff}}_{S}\left(s_{i},q_{i},\varepsilon \right)=\left\{\begin{array}{cc} 0, & 2\cdot |s_{i}\cap q_{i}\left|>\varepsilon \cdot (\right|s_{i}\left|+\right|q_{i}\left|\right),\\ 1, & \mathrm{otherwise}. \end{array}\right.$$

The parameter ε∈[0,1] allows us to consider an incomplete matching between the sets of active sensors, since the data captured through sensor devices tend to be noisy. The parameter determines the ratio of matching active sensors from both vectors to the sum of active sensors in both vectors, which is needed to call an agreement.

### 4.2. Comparing Sequences of Activities

Using ADL recognition techniques, sensor data are transformed into an activity sequence. We consider daily activity sequence $\vec{a}$ as a vector of activities from the finite alphabet A in successive time slots.

(5) $$\vec{a}=\left[a_{1},\ldots ,a_{n}\right],\;\;\;\;\;a_{i}\in A.$$

We call this a daily activity vector. Since the position in the sequence conveys time information, the difference between two positions defines a duration. As in the case of sensors, the dimension of the vector *n* depends on the time scale.

#### 4.2.1. Entropy

Some individuals, if they have a routine, perform their daily activities in a predictive way. Others have very diverse timelines. Shannon entropy can be used to measure the uncertainty of activity at time slot *i*. It is calculated using:

(6) $$h_{i}=-\sum_{j=1}^{A}p\left(a_{i,j}\right)\cdot \log_{2}p\left(a_{i,j}\right),\;\;\;\;A=\left|A\right|,$$

where $p(a_{i,j})$ denotes the probability of activity $a_{j}$ at time slot *i*. Entropy is 0 or close to 0 if the activity at the considered time slot is predictable, with the probability close to 1. Entropy is close to its maximum, which is $\log_{2}A$, if all activities are equiprobable.

In Equation (6), the activity at a considered time slot is selected independent of previous activities. However, in real-life scenarios, activities are not independent. If we consider that the selection of an activity at a considered time slot *i* is dependent on the activity at the immediately preceding time slot i−1 (i.e., a first-order Markov source), the conditional entropy is calculated as:

(7) $$h_{i}^{*}=-\sum_{k=1}^{A}p\left(a_{i-1,k}\right)\cdot \sum_{j=1}^{A}p\left(a_{i,j}|a_{i-1,k}\right)\cdot \log_{2}p\left(a_{i,j}|a_{i-1,k}\right),\;\;\;\;A=\left|A\right|,$$

where $p(a_{i-1,k})$ denotes the probability of activity $a_{k}$ at time slot i−1 and $p(a_{i,j}|a_{i-1,k})$ is the conditional probability of activity $a_{j}$ at time slot *i* if the activity $a_{k}$ was performed at time slot i−1.

Using entropy, we estimate how difficult it is to predict the daily activity vector for a given resident.

#### 4.2.2. Generalized Hamming Distance

The simple Hamming distance between two daily activity vectors $\vec{a}$ and $\vec{b}$ is the number of positions in which the two vectors of daily activities are different (see Equation (1)), where the difference function is defined as:

(8) $${\mathrm{diff}}_{A}\left(a_{i},b_{i}\right)=\left\{\begin{array}{cc} 0, & a_{i}=b_{i},\\ 1, & a_{i}\neq b_{i}. \end{array}\right.$$

We denote the Hamming distance based on Equation (8) with H1.

A generalization will allow for state-dependent costs of mismatching. The generalized Hamming distance is defined as the sum of activity-dependent position-wise mismatches between two daily activity vectors by using the difference function:

(9) $${\mathrm{diff}}_{G}\left(a_{i},b_{i}\right)=\left\{\begin{array}{cc} 0, & a_{i}=b_{i},\\ cost, & a_{i}∼b_{i},\\ 1, & a_{i}\neq b_{i}. \end{array}\right.$$

Here, cost is a fixed value in the interval [0,1], and a∼b denotes adjacency of *a* and *b*, which means that the activities are different, but a transition exists from activity *a* to activity *b* or from activity *b* to activity *a*, i.e., the two activities are consecutive to each other at least once in the dataset. A typical example would be the activity pair “meal preparation” and “eating”.

The last option, a≠b, denotes that activities *a* and *b* are neither the same nor adjacent. We denote the Hamming distance based on Equation (9) with H2.

Interleaved or concurrent activities may occur. If there is a possibility of two concurrent activities, the generalized Hamming distance must take all possible transitions into account. We now use $a_{i}$ and $b_{i}$ as a notation for sets of concurrent activities at time slot *i*. We limit the number of concurrent activities to two. The sets can now have one or two elements, e.g., $a_{i}=\left\{a_{i,1}\right\}$ or $a_{i}=\left\{a_{i,1},a_{i,2}\right\}$.

The generalized Hamming distance for this case uses the difference function:

(10) $${\mathrm{diff}}_{G*}\left(a_{i},b_{i}\right)=\left\{\begin{array}{cc} 0, & a_{i}=b_{i},\\ cost, & a_{i,1}∼b_{i,1}\;\wedge |a_{i}\left|=\right|b_{i}|=1,\\ cost, & \left(a_{i,1}∼b_{i,1}\vee a_{i,2}∼b_{i,1}\right)\wedge |a_{i}\left|=2\wedge \right|b_{i}|=1,\\ cost, & \left(a_{i,1}∼b_{i,1}\vee a_{i,1}∼b_{i,2}\right)\wedge |a_{i}\left|=1\wedge \right|b_{i}|=2,\\ cost, & \left(a_{i,1}∼b_{i,1}\vee a_{i,1}∼b_{i,2}\vee a_{i,2}∼b_{i,1}\vee a_{i,2}∼b_{i,2}\right)\wedge |a_{i}\left|=\right|b_{i}|=2,\\ 1, & a_{i}\neq b_{i}. \end{array}\right.$$

In Equations (9) and (10), the costs of mismatches (denoted as cost) can be fixed, or they could be derived from the observed transition rates. The probability of transition from activity *a* to activity *b* in the sequence is estimated as:

(11) $$p\left(b|a\right)=\frac{\sum_{j=1}^{d}C_{j}\left(a\to b\right)}{\sum_{j=1}^{d}C_{j}\left(a\right)},$$

where *d* denotes the number of observed days, $C_{j}\left(a\to b\right)$ counts the number of transitions from activity *a* to activity *b* in the daily activity vector of day *j*, and $C_{j}\left(a\right)$ counts the number of transitions from activity *a* to any other activity in the vector for day *j*. The symmetrical cost is defined as:

(12) cost=1−0.5·p(a|b)−0.5·p(b|a).

We denote the Hamming distance with costs from Equations (11) and (12) with H3.

The Hamming distance is symmetrical $(H\left(\vec{a},\vec{b}\right)=H\left(\vec{b},\vec{a}\right))$, and the symmetry is preserved in generalized Hamming distances H2 and H3. The similarity measure expresses the similarity between two vectors on a scale from 0 to 1. For the Hamming distance, it is defined as:

(13) $$sim_{H}\left(\vec{a},\vec{b}\right)=1-\frac{H(\vec{a},\vec{b})}{n}.$$

#### 4.2.3. Levenshtein Distance

Daily activity vectors could be compared as sequences of activities irrespective of their duration. The Levenshtein distance measures the distance in this sense. The Levenshtein distance between two daily activity vectors $\vec{a}$ and $\vec{b}$ is given in Equation (2). In our experiments, we set $cost_{I}=cost_{D}=1$ and $cost_{S}=2$.

The similarity measure, defined with the Levenshtein distance, is:

(14) $$sim_{L}\left(\vec{a},\vec{b}\right)=1-\frac{L(\vec{a},\vec{b})}{\max(|\vec{a}|,|\vec{b}|)}.$$

The Hamming distance can only be applied to sequences of equal length. On the contrary, the Levenshtein distance can be computed between sequences of different lengths. By shrinking the activity sequence to transitions between activities, the time span of each activity is lost. Where the timing of activities is crucial, Hamming distance should be used. Where it is less important, the Levenshtein distance could be more appropriate.

### 4.3. Clustering

Based on the above-described distance metrics of sensor or activity data, we can form a distance matrix for all the days in our datasets. Hereafter, the days in the datasets are our data points for clustering, which is used to divide the data points into partitions. Since the data points are not in a vector space, we cannot calculate means for partitions. Therefore, clustering is based on medoids instead. A medoid is a representative data point, and serves as the “center” of the partition to which distances from other data points are used.

Clustering is performed using the Partition Around Medoids (PAM) algorithm [38], which works in two phases. In the first phase, a predetermined number of elements *k* from the set is randomly selected as possible medoids—one for each cluster. All data points are then associated with the closest medoid candidate, and a cost function is calculated. The cost function is the sum of distances from all data points in the dataset to their medoids.

In the second phase, medoids are swapped with other data points, and the cost function is recalculated. This swap is repeated for pairs of metoids and non-metoid data points. Only the swap, which results in the best new cost function value, is then applied for the next iteration. Iterations are repeated as long as the cost function improves.

The clustering algorithm results are *k* partitions, each containing a number of the data points, one of which is the medoid. For a better graphical representation, the distance matrix can be rearranged so that data points belonging to the same partitions form consecutive rows and columns in the matrix.

We consider two different clusterings to be similar if the data points are clustered in similar partitions. In order to determine such similarities, we calculate the frequently used Rand index. This index is calculated as a value between 0 and 1, where higher values mean higher similarity and lower values mean lower similarity. The value of the Rand index is 1 if and only if the two partitionings are identical.

However, it was shown that typically values of the Rand index are in an interval close to 1. Even in the case of statistical independence, the index values can be rather high, and must, therefore, be interpreted carefully [39].

The Rand index is calculated using the formula:

(15) R=Ns+Ndm2,

where $N_{s}$ is the number of data-point pairs that are in the same partition in both partitionings, $N_{d}$ is the number of data-point pairs that are in different partitions in both partitionings, and *m* is the total number of data points in the dataset.

## 5. Experiments

### 5.1. Data Sets

The problem tackled in our research is the discovery of daily activity patterns from vectors of active sensors and vectors of activity sequences. Currently, many sensor-based datasets for ADL recognition are available to end-users and researchers [21]. However, to the best of our knowledge, there is no dataset with ADL recognition results available directly. For this reason, the main guiding principle in the selection of the dataset were the published ADL results (classification accuracy, F-measure), which ensures that the activities can be identified correctly from the sensor readings in the dataset.

The experiments were performed on two different datasets. The Kasteren dataset (http://casas.wsu.edu/datasets/kasterenDataset.zip, accessed on 31 May 2021) was recorded in an apartment with three rooms, where one 27-year old male lived. The dataset was annotated using a handwritten diary of activities made by the resident. At certain time intervals, two activities were annotated as concurrent (for example, ”use toilet” and ”go to bed”). ADL recognition accuracy of 95.6 % was reported for the Kasteren dataset [40]. The CASAS center (http://casas.wsu.edu/datasets/, accessed on 31 May 2021) collected several datasets of sensor and activity data [41]. We selected the CASAS 11 dataset. This dataset was collected in an apartment with two residents with spontaneous activities and no predetermined scenarios. It also has concurrent activities. A comparable accuracy for the Kasteren and CASAS datasets was reported in [42]. Several other studies reported high recognition accuracy in CASAS datasets with daily living data. An overview of these studies can be found in [21]. Details of the datasets used in experiments are collected in Table 2. We followed the rule that datasets should be of comparable sizes, and used only the first 30 days of the CASAS 11 dataset.

Since the CASAS 11 dataset has two residents and later in the paper, we will analyze the activities for both residents separately, we will refer to a total of three datasets.

### 5.2. Data Preprocessing

We considered only binary sensor data and, therefore, excluded non-binary sensor data, such as temperature, electric power consumption, etc. In the CASAS 11 dataset, additionally, we had to make some minor corrections (e.g., sensor value “OF” is replaced with “OFF,” the year 22009 was corrected to 2009, etc.).

All datasets were then reformatted into a new format—a text file, where each line corresponds to one time slot (a second), and all binary sensor and activity data are written in columns with numeric values 0 and 1. Timestamps for events (changes in sensor value or activity transitions) were rounded to the nearest second, where needed.

**Figure 2:**
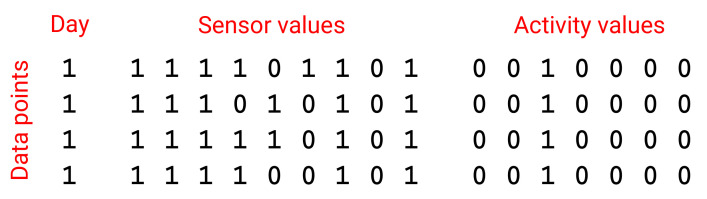
Excerpt from the preprocessed dataset. The first column denotes the day in the dataset, the following columns denote sensor values (one column per sensor), and the last columns denote activity values (one column per activity). Each line represents one data point and corresponds to one time slot. Value 1 denotes active sensor or present activity.

An examination of the daily activities in both datasets revealed that the residents were performing different activities on different days at midnight. In order to have the same activity at the start and end of each day—sleep—we decided to shift the start. Therefore, we decided to start a day in our experiments at 4 a.m. on one calendar day and end the day at 4 a.m. on the next calendar day. The format of the preprocessed datasets is presented in Figure 2.

Due to this shift, we disregarded the first 4 h of the first day from all datasets. In the CASAS 11 datasets, we then also used 4 h from the first three days to obtain the full 30 periods of 24 h. In the Kasteren dataset; however, there were no more data for the following day. The last day in the dataset ended with no activity at all, indicating that the resident was away for the night. We decided to extend this state for another 4 h to complete the reformatted dataset.

We found that in both datasets, two activities could occur at the same time. In the Kasteren dataset, the activity ”use toilet” can occur during the activities ”prepare dinner” and ”go to bed,” which—judging from the data—also means staying in bed and sleeping. Similarly, in the CASAS 11 datasets, concurrent activities are possible for each of the two residents, e.g., ”eating” and ”watching TV”.

We were interested in the residents’ daily habits. Can we define their routine directly from sensor data, or do we need ADL recognition first? To this end, we performed two types of transformations from the new file format. We created a file where each line corresponded to active sensors in one day. In the second file, each line corresponded to activities performed on one day. In both files, each column corresponded to one second. These files were then used to perform distance metric calculations and for a graphical representation of activity patterns.

### 5.3. Results and Discussion

#### 5.3.1. Entropy

**Figure 3:**
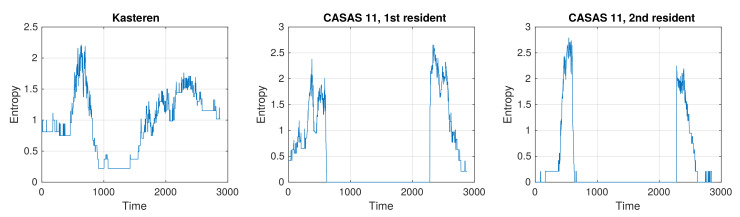
The entropy of activities at different times of the day for the Kasteren dataset, the first resident of the CASAS 11 dataset, and the second resident of the CASAS 11 dataset. A day starts at 4 a.m. on one calendar day and ends at 4 a.m. on the next calendar day. In the CASAS 11 dataset, entropy was calculated separately for each of the two residents. It was calculated every half minute.

First, we were interested in how uncertain the activities were at different times. Entropy plots for all datasets were calculated (see Equation (6)) and are given in Figure 3. Entropy was calculated every half minute. For this purpose, we resized the daily activity vectors from the dimension *n* = 86,400 to the dimension n=2880. In this case, *i* denotes the *i*-th time slot with the duration of half a minute. Data for the time slots in the resized vector were obtained by merging data from an interval of time slots in the original vector.

An activity is marked as present if it is present in at least one time slot within the corresponding interval. Entropy is always lower than 2.8 bits. In the Kasteren dataset, the average entropy is 0.95 bits, whereas in the CASAS 11 datasets, it is 0.51 and 0.33 bits. Entropy between the Kasteren and CASAS datasets cannot be compared directly quantitatively, as the CASAS datasets have more activities than the Kasteren dataset.

In the Kasteren dataset, uncertainty at night was caused by the activity ”use toilet”, surrounded by the activity ”go to bed.” Uncertainty in the morning resulted from occasionally skipping the activities ”prepare breakfast” or ”take shower.” In the middle of the day, the entropy falls to a small value as the resident has almost always left home. Uncertainty in the evening is a consequence of the activities ”prepare dinner,” ”get drink,” ”use toilet,” and ”take shower,” which were not taken consistently.

In both CASAS 11 datasets, we can see a long period with the entropy equal to zero. This indicates that, at this time, the activity is always the same. Each day, both residents left home for a long period of time, as is evident from the the activity sequence ”leave home,” ”no activity,” and ”enter home.” On certain days, activity "leave home" or activity "enter home" is missing, but its occurrence is evident from the previous activity or the following activity.

**Figure 4:**
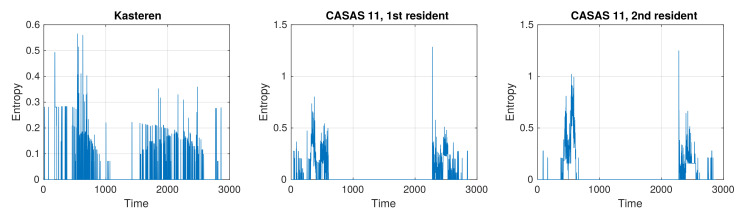
The conditional entropy of activities at different times of the day for the Kasteren dataset, the first resident of the CASAS 11 dataset, and the second resident of the CASAS dataset.

Plots in Figure 4 show the conditional entropy (see Equation (7)). In all plots, it is lower than 1.3 bits. Conditional entropy is always lower than unconditional entropy. As expected, the activities in adjacent time slots are not independent. All calculated entropies are also much lower than their upper limits, being $log_{2}7=2.8\;$ bits for the Kasteren dataset and $log_{2}13=3.7\;$ bits ($log_{2}12=3.58\;$ bits) for the CASAS 11 dataset, which confirms that activities are not selected randomly. All observations for unconditional and conditional entropy show patterns of normal behavior of the resident.

Given that the entropies for both residents in the CASAS 11 datasets are higher than the entropy in the Kasteren dataset despite having more possible activities, we can conclude that the residents in the CASAS 11 datasets are more consistent in their daily activities.

#### 5.3.2. Distances between Daily Activity Vectors

From the reformatted datasets, the distances between days were calculated based on the metrics described in Section 4.1 and Section 4.2. We chose to use distances rather than similarities. This decision does not influence the final results, as they are equivalent. The distances can be calculated based on sensor data or daily activity vectors.

First, we were interested in activity vectors. Hamming distance and Levenshtein distance were examined. When using the Hamming distance, the activities of each day are presented with a daily activity vector of constant size *n* = 86,400, where one component in the vector corresponds to one second. For the Levenshtein distance, the vector sizes are smaller and diverse, where one component in the vector corresponds to one activity regardless of its duration.

**Figure 5:**
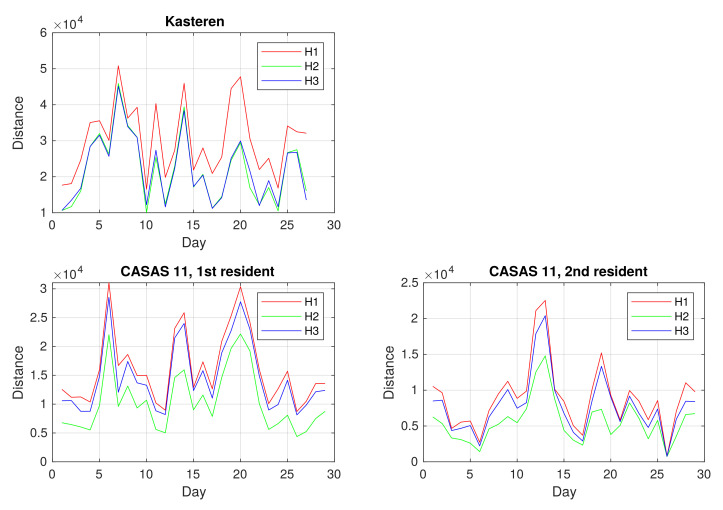
Hamming distances between daily activity vectors for consecutive days for the Kasteren dataset, the first resident of the CASAS 11 dataset, and the second resident of the CASAS dataset.

We were interested in the distances between consecutive days in the datasets. All three types of Hamming distance between activity vectors of consecutive days for all datasets are shown in Figure 5. In the Kasteren dataset, distances ranged from 10,000 to 50,000, whereas, in the CASAS 11 datasets, they were between 0 and 30,000. As data points correspond to seconds, the distance 50,000 means that activities in two daily vectors do not match for almost 14 h. Distance 0 corresponds to two days, on which the second resident in the CASAS 11 dataset left home. As expected, H3 distances are shorter than H1 distances and larger or equal to H2 distances, as H2 and H3 use *cost* values smaller than 1. Our general observation is that the Hamming distance between activity vectors of consecutive days can vary a great deal, and we cannot infer unusual behavior of the resident from them.

In Table 3, we collected the average values for all Hamming distances between consecutive days for all datasets. We can see that the average distances are quite large, even using a generalization of the Hamming distance. The large average distances between consecutive days show that the behavior of residents changes from day to day.

#### 5.3.3. Clustering of Daily Activity Vectors

In the continuation of our study, we were interested to discover if we could define partitions of common daily activity patterns by grouping daily activity vectors having shorter distances. Thus, we wanted to identify days with similar behavior of the residents. For further experiments, we used the metrics H1 and H3. The graphs in Figure 5 show similar behavior for all three metrics.

**Figure 6:**
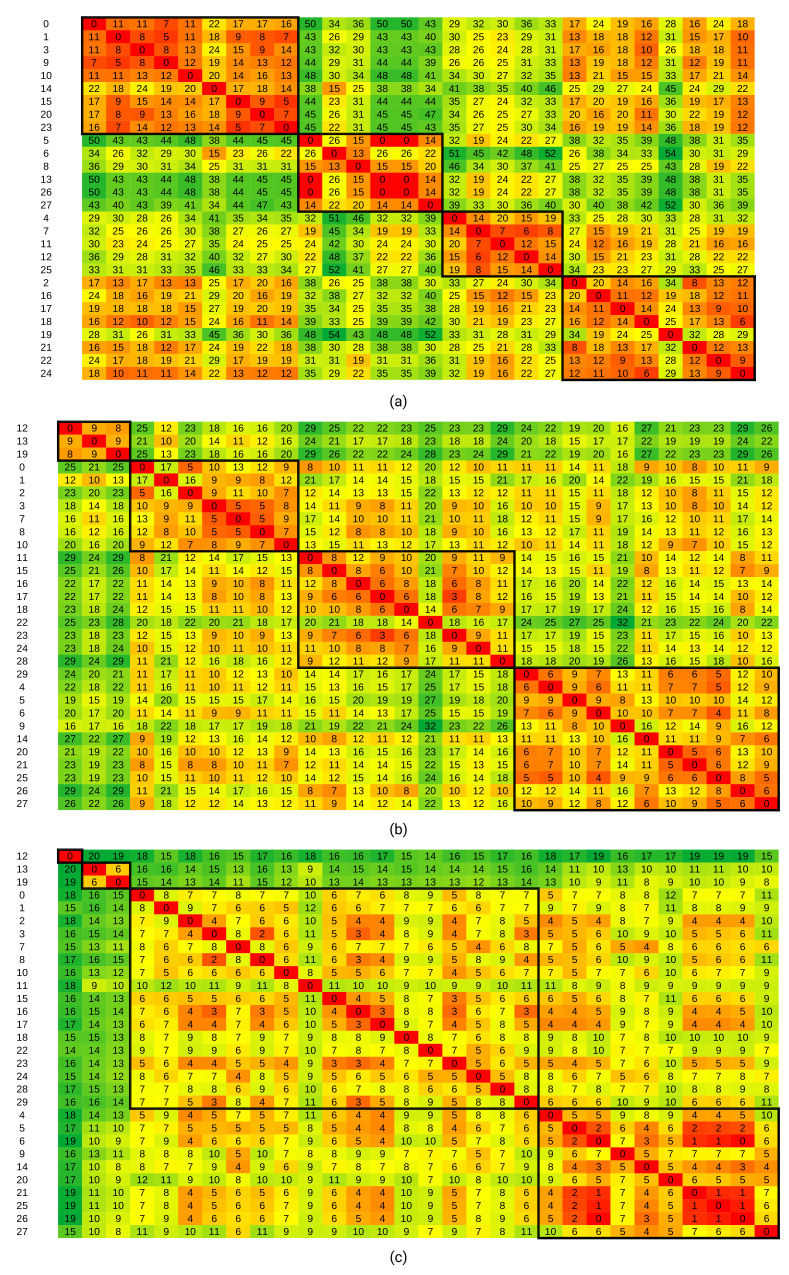
Distance matrices of the H3 metric between daily activity vectors for (**a**) Kasteren dataset, (**b**) The first resident of the CASAS 11 dataset, and (**c**) The second resident of the CASAS dataset. Values are in thousands. The background color shows gradient changes in values, with red tones indicating low values and green tones indicating high values.

We formed distance matrices containing the distances, taken pairwise, between activity vectors for all days. These matrices were used for clustering, which was performed as described in Section 4.3. The resulting matrices, in which activity vectors were reorganized according to the obtained partitions, are given in Figure 6.

**Figure 7:**
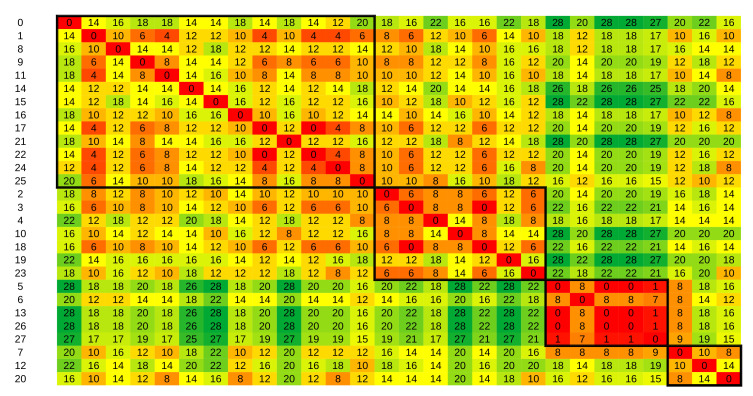
Distance matrix of the Levenshtein metric between daily activity vectors for the Kasteren dataset. Values are in thousands. The background color shows gradient changes in values, with red tones indicating low values and green tones indicating high values.

**Figure 8:**
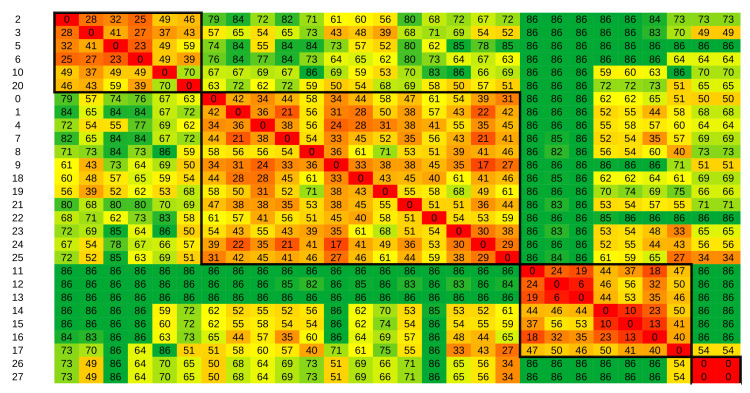
Distance matrix based on sensor data with ε=0.7 for the Kasteren dataset. Values are in thousands. The background color shows gradient changes in values, with red tones indicating low values and green tones indicating high values.

In Figure 6, Figure 7 and Figure 8, the color scheme indicates distances, with green tones representing large distances and red tones representing small distances. The numeric distance values are in thousands. Bolted borders enclose distances between data points from the same partition. The numbers on the left side denote consecutive numbers for the days in the dataset (starting with 0). Figure 6, Figure 7 and Figure 8 were obtained using the H3 metric. Similar figures can be obtained using the H1 metric.

From Figure 6, partitions can be seen clearly. For example, in the Kasteren dataset, we see that distances within the first partition (top left to bottom right) are significantly smaller than the distances between days from the first partition and days from the second or third partitions. Distances between days from the first partition and days from the fourth partition are smaller than distances between other combinations of partitions, but still larger than distances within the first partition. This observation indicates that the first and fourth partitions contain daily activity vectors with some degree of similarity. We can also extend such interpretations to other pairs of partitions in all three datasets.

Graphical depictions of the distances within partitions show that a four partition solution achieves a balance between internal partition distances and the interpretability of the partitions.

The same procedure can be repeated using other distance metrics. However, we find that partitions are most clearly distinguishable using H3, as shown when comparing Figure 6a with Figure 7, in which the Levenshtein distance was used. Although we can still recognize partitions, aside from the third partition, they are not as distinguished as in the clustering with the H3 metric. The Rand index between these two clustering results was 0.67, indicating a very loose agreement between them.

In the next set of experiments, we performed clustering based on the distances from sensor data alone (see Equations (1) and (4)). If we obtain comparable clustering results, ADL recognition would not be necessary to investigate residents’ daily living.

The resulting distance matrix is presented in Figure 8. These results were obtained by setting the parameter ϵ to 0.70. Similar results were obtained with values from 0.50 to 0.90. Although partitions were now well distinguishable, the clustering result was not in agreement with the clustering based on activity data—the Rand index between them was 0.60. This result indicates that clustering should be done on activity data and not on sensor data. Therefore, we can argue that ADL recognition is necessary.

Although ADL recognition does not give perfect results, an accuracy of 95%, if it is based on time-slots, would give a Hamming distance H1 of 4320 between the recognition results and the reference data. The metrics H2 and H3 would be even lower. Since the typical distances between days and clusters are significantly greater, we can argue that such an ADL recognition accuracy may be sufficient for the purpose of clustering daily activity vectors, although further studies would be necessary to confirm this.

The average Hamming distances H1 and H3 between days are given in Table 4 and Table 5. In the first row, we have the average over the distances between all possible pairs of days. The following rows show distances of all possible pairs within a given partition, and the last row gives the average over all four partitions.

Missing data indicates only one day in the given partition. The data show that distances within partitions are significantly smaller than over all days, confirming that our clustering method indeed generates partitions with similar days according to the H1 and H3 metric for activities.

However, the ratio of the distances is larger for H3 than H1, giving a better differentiation between partitions. When using the H1 metric, we obtain more partitions with a single day. Therefore, we present our research results using the H3 metric.

#### 5.3.4. Graphical Presentation of Daily Activity Vectors

**Figure 9:**
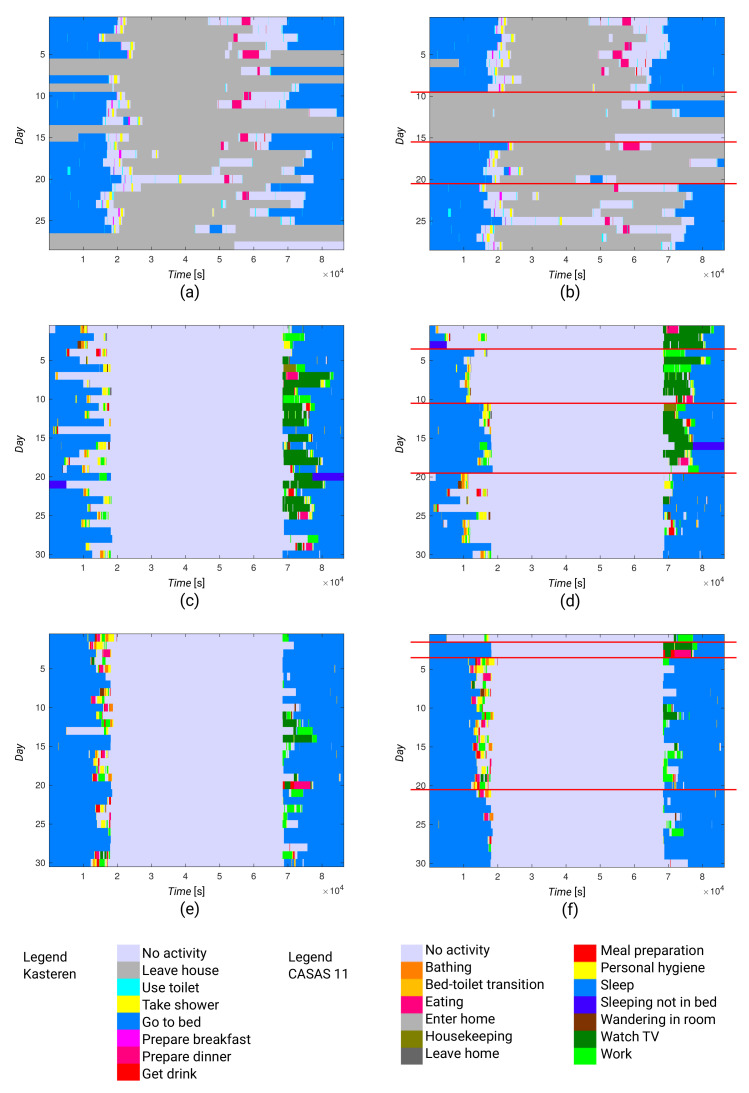
Daily activity representations of the resident in the (**a**) Kasteren dataset, consecutive days; (**b**) Kasteren dataset, partitioned on daily activity vectors; (**c**) CASAS 11 dataset, first resident, consecutive days; (**d**) CASAS 11 dataset, first resident, partitioned on daily activity vectors; (**e**) CASAS 11 dataset, second resident, consecutive days; and (**f**) CASAS 11 dataset, second resident, partitioned on daily activity vectors.

Having partitions, we were interested in activity patterns that were common to daily activity vectors in the same partition. We made a graphical representation of the activity clusters so that we could obtain a more intuitive view of them. Activity patterns are evident from Figure 9, where we compare the daily activity vectors for consecutive days with the daily activity vectors grouped according to partitions.

By comparing the daily activity vectors for consecutive days (Figure 9a,c,e), we can see dissimilarities between vectors for consecutive days. This observation is consistent with the high values in Figure 5 and Table 3.

On the contrary, we can examine the graphical presentation for the partitioned daily activity vectors. For example, in the Kasteren dataset (Figure 9b), we can see similarities between vectors within partitions. We see that the second and third partitions contain vectors that are very dissimilar to the vectors in the other two partitions. In the second partition, the early hours do not contain any activity (light blue), which might mean that the resident was not in the apartment at this time.

In the third partition, this same lack of activities is shown in the evening and the night hours. The differences between the first and fourth partitions are smaller. However, in the first partition, we can see more activities in the early evening hours (time between 50,000 and 60,000) and earlier transition to bed (green) than in the fourth partition. These observations are consistent with our earlier interpretation of the distance matrix in Figure 6a.

Similarly, we can examine the graphical presentation for the partitioned daily activity vectors for both residents in the CASAS 11 dataset (see Figure 9d,f). However, we can also see that both residents in this dataset had a more consistent daily routine than the resident in the Kasteren dataset.

**Figure 10:**
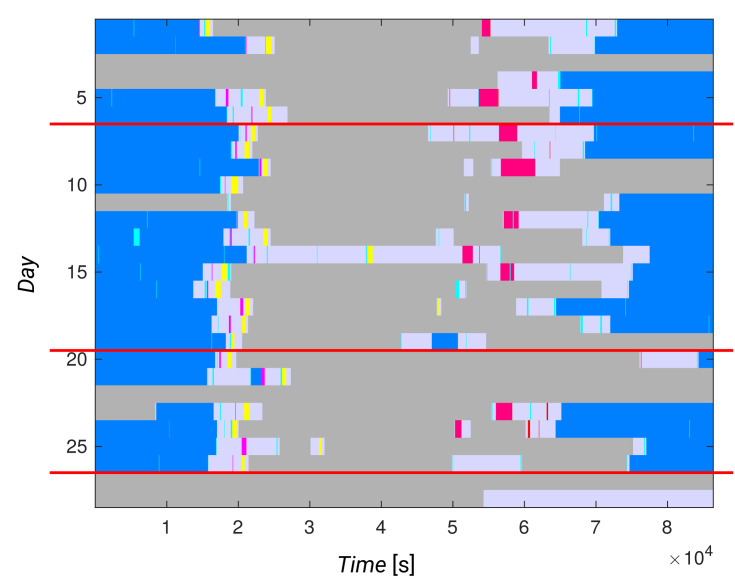
Daily activity representations of the resident in the Kasteren dataset, partitioned according to the clustering results based on sensor data.

In Figure 10, daily activity vectors from the Kasteren dataset are clustered according to sensor data (see the distance matrix in Figure 8). The Figure shows that the daily activity vectors within partitions are far more varied than the results from clustering based on activity data, showing the need for activity recognition.

From Figure 9f, we can easily recognize a single day with unusual behavior in the first partition when compared to the other days. Thus, we may say that such a graphical representation can also be used in practice. It may be useful for a physician or caregiver, as they may be able to look quickly at a resident’s normal behavior, their recent behavior, and recognize changes without the need for a lengthy conversation.

## 6. Conclusions

Elderly people often have a regular daily routine that gives structure and a natural flow to the day. Deviations from this routine may indicate health problems. On the other hand, the everyday life of the younger population often seems unpredictable and disorganized. In this paper, we have shown that, even for younger people, we can identify common patterns regarding how individuals sequence their everyday activities during the day. We define a framework that involves a definition of a generalized Hamming distance to quantify the degree of similarity between each pair of daily activity sequences in the dataset, and then to use all of this pairwise information stored in a distance matrix to identify partitions of similar daily patterns. The obtained partitions identify the normal routines of the residents.

This research was performed on annotated data from the original datasets. In the future, a research interest may be in comparing the clustering results from annotated data and different automatic recognition results. For this purpose, a dataset with publicly available reference recognition results would be useful.

Our future work will focus on a method to detect abnormal signs in a daily activity sequence by comparing it with recognized partitions of normal routines. Having such a method will also provide valuable information in the current outbreak of Coronavirus (COVID-19) disease [43]. Detecting the deterioration of ADL performance of COVID-19 patients after the acute phase of infection will contribute toward identifying the need for rehabilitation. On the other hand, investigating the routines of a person’s life across days also provides valuable information for intelligent recommendation systems for healthy living. For example, lack of sleep affects the ability to do everyday activities. Too many snacks can also lead to health issues. Both cases can be identified by the framework described in this paper.

## Figures and Tables

**Table 1:** Studies related to our work.

Refs	Aim	Data or Methods Used
[21]	review	ADL datasets
[15]	ADL recognition	Naive Bayes classifier
[8,16,17]	ADL recognition	HMM
[3]	ADL recognition	Support Vector Machines
[6,19,20]	ADL recognition	neural networks
[31]	ADL recognition	clustering and classification
[4]	ADL recognition	clustering
[30]	review	alignment based similarity measures
[28]	review	similarity measures and distances
[26,27]	life trajectories study	sequence comparison
[25]	analysis of biological sequences	sequence comparison
[7]	ADL definition	clustering
[34]	discovering ADL patterns	similarity adapted to selected features
[32]	discovering ADL patterns	neural network model wit ADL features
[33]	discovering ADL patterns	HMM
	and anomaly detection	
[35]	anomaly detection	numerical distances
[36]	anomaly detection	numerical Euclidean distance
[37]	anomaly detection	Channel State Information

**Table 2:** Datasets used in experiments.

Dataset	Kasteren	CASAS 11
Occupancy	1 resident	2 residents
Capture	28 days	232 days
Number of sensors	14	88
Number of binary sensors	14	82
Number of activities	7	13 + 12
Maximum no. of concurrent activities	2	2

**Table 3:** Average Hamming distances between consecutive days.

Dataset	H1	H2	H3
Kasteren	30,341.63	21,888.31	22,134.49
CASAS 11, first resident	16,218.69	10,378.21	14,588.89
CASAS 11, second resident	8910.83	5555.45	7720.90

**Table 4:** Average Hamming distances H1 between all days and days in the same partitions.

Dataset	Kasteren	CASAS 11, First Resident	CASAS 11, Second Resident
All days	33,704.20	16,354.60	9232.97
Partition 1	19,629.49	14,408.00	/
Partition 2	15,353.48	8643.72	7252.16
Partition 3	19,380.11	/	7547.24
Partition 4	/	14,219.90	7382.67
Partition average	18,121.03	12,423.87	7394.02

**Table 5:** Average Hamming distances H3 between all days and days in the same partitions.

Dataset	Kasteren	CASAS 11, First Resident	CASAS 11, Second Resident
All days	25,881.38	14,335.95	8092.51
Partition 1	12,896.39	8640.04	/
Partition 2	14,480.30	9345.59	5611.50
Partition 3	13,203.07	10,680.56	6641.08
Partition 4	16,308.99	9300.99	4235.39
Partition average	14,222.19	9491.80	5495.99

## Data Availability

Publicly available datasets were analyzed in this study. This data can be found here: http://casas.wsu.edu/datasets/ (accessed on 31 May 2021) and http://casas.wsu.edu/datasets/kasterenDataset.zip (accessed on 31 May 2021).
